# Cellular Processing of the ABCG2 Transporter—Potential Effects on Gout and Drug Metabolism

**DOI:** 10.3390/cells8101215

**Published:** 2019-10-08

**Authors:** Orsolya Mózner, Zsuzsa Bartos, Boglárka Zámbó, László Homolya, Tamás Hegedűs, Balázs Sarkadi

**Affiliations:** 1 Institute of Enzymology, Research Centre for Natural Sciences, Magyar Tudosok krt. 2, 1117 Budapest, Hungary; 2 MTA-SE Molecular Biophysics Research Group, Hungarian Academy of Sciences, Tűzoltó u. 37-47, 1094 Budapest, Hungary; 3 Department of Biophysics and Radiation Biology, Semmelweis University, Tűzoltó u. 37-47, 1094 Budapest, Hungary

**Keywords:** ABC transporters, ABCG2 multidrug transporter, ABCG2 trafficking, gout, drug metabolism

## Abstract

The human ABCG2 is an important plasma membrane multidrug transporter, involved in uric acid secretion, modulation of absorption of drugs, and in drug resistance of cancer cells. Variants of the ABCG2 transporter, affecting cellular processing and trafficking, have been shown to cause gout and increased drug toxicity. In this paper, we overview the key cellular pathways involved in the processing and trafficking of large membrane proteins, focusing on ABC transporters. We discuss the information available for disease-causing polymorphic variants and selected mutations of ABCG2, causing increased degradation and impaired travelling of the transporter to the plasma membrane. In addition, we provide a detailed in silico analysis of an as yet unrecognized loop region of the ABCG2 protein, in which a recently discovered mutation may actually promote ABCG2 membrane expression. We suggest that post-translational modifications in this unstructured loop at the cytoplasmic surface of the protein may have special influence on ABCG2 processing and trafficking.

## 1. Overview of Cellular Processing and Trafficking of Plasma Membrane ABC Proteins—Role in Diseases

Plasma membrane integral membrane proteins, from the site of their synthesis in the endoplasmic reticulum (ER) surface and glycosylation in the Golgi apparatus, travel to the plasma membrane by vesicles containing the coatomer complexes, from the trans-Golgi network through early endosomes. These mechanisms are clearly present for plasma membrane resident ABC (ATP-binding cassette) proteins and determine at a large extent their levels of expression and function [1].

There are two major quality control mechanisms affecting the plasma membrane proteins: one in the ER and Golgi (a “central quality control”), and a second one at or near the plasma membrane (“peripheral quality control”). In the central control mechanism, proteins that are not properly folded (and usually only core proteins) are directed towards the proteasomes, while properly folded transmembrane proteins obtain their final glycosylation in the Golgi apparatus and travel to the plasma membrane through a vesicular transport mechanism. The peripheral quality control involves endosomal recycling, transfer to the late endosomes, and either recycling or an eventual lysosomal degradation. The two quality control pathways are interconnected, so misfolded transmembrane proteins may travel to the late endosomes directly from the ER or Golgi, while plasma membrane localized proteins may be partially degraded by the proteasomes. If the protein degradation system is overwhelmed, specific aggresomes, accumulating partially degraded proteins, are formed near the nuclei within the cells [2,3,4,5].

Ubiquitination and de-ubiquitination play major roles in both steps of quality control; transmembrane proteins are ubiquitinated at specific lysine residues [6]. These modifications usually occur in the cytosolic and often unstructured domains of these proteins, serving as sorting signals for the associating regulatory or cargo molecules. In the central quality control, ubiquitin directly acts as a signal for degradation in the proteasomes, and the peripheral quality control may also include a ubiquitin-dependent regulation of recycling or lysosomal degradation [4,7]. In fact, these quality control mechanisms have numerous regulating protein participants at each step of the quality control.

In many cases these general routes of trafficking are complemented by specific pathways directing ABC proteins to specific membrane compartments (e.g., the apical or basolateral membranes of polarized cells). Unconventional trafficking routings include plasma membrane delivery through a rab11-positive endosomal pool [8] or via a Golgi reassembly stacking protein (GRASP)-dependent pathway that bypasses the Golgi [9]. It has even been proposed that some ABC proteins are required to undergo rapid internalization and transcytosis for functional maturation [10]. The central and peripheral quality control mechanisms in the cases of several plasma membrane ABC transporters involved in major human diseases are being increasingly explored and understood [11,12].

Failure in the trafficking of ABC membrane proteins is often caused by missense mutations or small deletions, affecting folding or cellular processing. The improper folding and processing lead to reduced levels of the protein in the target membrane compartment, thus altering the function and subsequently leading to disease development. A widely studied example for such a failure is the folding and trafficking problem of the ABCC7 protein (cystic fibrosis transmembrane conductance regulator, CFTR) caused by the most frequent mutations in the disease, cystic fibrosis (CF).

Cystic fibrosis is the most frequent lethal recessive hereditary disease, affecting around 1 in 2500 newborns of Caucasian origin. The CFTR (ABCC7) protein functions as an anion channel in the apical membrane of epithelial cells in several tissues by conducting Cl^−^ and bicarbonate according to the electrochemical gradient [13]. More than 1900 different mutations have been reported so far in the *CFTR* gene. These can be categorized into 6 classes, according to their effect on the protein [14,15]. The most frequent variant of ABCC7 is ΔF508. This mutant protein displays defective trafficking and low abundance in the plasma membrane. The mutant ΔF508-CFTR is partially functional but is mostly retained in the ER and degraded through the proteasomal pathway. Even in the case of the wild-type CFTR, central quality control removes a large fraction of the core glycosylated protein, and small changes in folding caused by various mutations induce rapid degradation and loss of function. It has been shown that the correct trafficking and function of these mutants can be achieved by the addition of small molecule correctors, including Lumacaftor/VX-809 [16] 4-phenylbutirate [7]. At present, a variety of therapeutically applicable, efficient corrector molecules are available to help patients with defective ABCC7 trafficking mutations [17].

Another example for the failure of trafficking of a plasma membrane ABC transporter involves pseudoxanthoma elasticum (PXE). This rare, recessive hereditary disease is caused by mutations in the ABCC6 organic anion transporter, resulting in ectopic calcification in the elastic tissues of the human body [18]. Several mutations in the *ABCC6* gene primarily affect the trafficking of the protein, while leaving the anion transport function of the transporter intact. The plasma membrane localization of trafficking mutant ABCC6 variants in the liver, their main site of expression, can be corrected by 4-PBA administration according to results observed in mice [19].

Mutations in the bile salt export pump (BSEP, ABCB11) the multidrug resistance protein 3 (MDR3, ABCB4) cause progressive familial intrahepatic cholestasis (PFIC) types II and III, respectively (severe cholestatic liver diseases appearing in early infancy [20,21]). ABCB11, normally transporting bile salts into the liver canaliculi, has several mutations that alter the membrane trafficking of the protein. These mutant variants undergo only core glycosylation and are predominantly located in the endoplasmic reticulum, although their transporter activity is preserved [22,23]. Again, chemical chaperones or trafficking modifiers may help to overcome the disease in the case of these types of mutations [24,25,26]. Similarly, mutations causing impaired trafficking of ABCB4, which normally functions as a phosphatidyl choline transporter in hepatocytes, can be rescued by pharmacological chaperones [27]. It has to be emphasized that for the selection of ABC transporter trafficking mutants available for potential correction therapy, functional assays, at least in vitro, but if possible, in vivo, also have to be performed. In the case of disease-causing mutants resulting in major functional defects, other therapeutic approaches have to be considered. The above mentioned ABC transporters and the related disease conditions are summarized in Table 1.

## 2. Regulation of Folding and Trafficking of the ABCG2 Multidrug Transporter

ABCG2 is a key protein in the extrusion of endo- and xenobiotics from numerous cell types and was shown to be involved in uric acid secretion, the modulation of drug absorption [28], as well as in the drug resistance of cancer cells [29]. The ABCG2 protein is typically located in tissue interfaces, such as the blood–brain barrier, placenta, and intestine, but it has a remarkable role in the protection of stem cells as well [30]. Interestingly, individuals with a lack of ABCG2 expression are basically healthy, and because ABCG2 is normally also expressed in the red blood cells, these individuals belong to a rare blood group called Jr negative (Jr^–^) [31].

There are numerous environmental or cellular metabolic factors that may influence the cell surface expression and activity of ABCG2. Transcriptional regulators of ABCG2 expression include numerous hormones and transcriptional factors, but cell differentiation and maturation are also important regulators [32,33,34,35,36,37,38]. In the human pluripotent stem cells, ABCG2 shows a variable expression level, depending on the culturing conditions, with cell differentiation into the different directions greatly modifying expression levels [39,40]. Interestingly, differentiation in the trophoblast direction strongly increases ABCG2 expression [41], while cardiac [42] or neuronal [43] differentiation decreases the expression of this transporter. Hepatic differentiation results in a transient decrease followed by a regain of ABCG2 expression [44]. In the case of pluripotent stem cells, an oxidative stress causes a reversible internalization of ABCG2 [45], with a currently unknown mechanism. Komori et al. [46] reported that hyperuricemia, presumably through a cellular oxidative effect, reduced the cell surface expression of ABCG2, while not affecting the general expression level of the protein. The oxidative stress effects may be connected to the finding that the PI3K-Akt-mTOR signaling pathway is involved in retaining the ABCG2 protein in the plasma membrane, while an inhibition of this pathway caused the appearance of the transporter in intracellular vesicles with enhanced recycling [47]. Since Akt phosphorylation is inhibited by cellular oxidative stress, the inhibition of this pathway may be involved in ABCG2 internalization. In addition, several drugs, inhibiting ABCG2 transport activity, were shown to induce reduction of the transporter at the cell surface and result in a lysosomal degradation of ABCG2 [48].

In addition to potential transcriptional and processing regulatory effects, in this paper we focus on the potential role of the hereditary genetic variants of ABCG2. It is remarkable that hundreds of missense variants have been found in the coding region of the *ABCG2* gene in humans, including relatively frequent polymorphisms (https://gnomad.broadinstitute.org/gene/ENSG00000118777). The two most common polymorphic missense variants are V12M (rs2231137, c.34G>A) and Q141K (rs2231142, c.421C>A). According to several studies, the V12M variant has no appreciable effect on the expression, localization, or function of the transporter [49,50,51]. Moreover, high-throughput studies and meta-analyses indicate that this variant may contribute to protection against gout [52,53,54]. In contrast, the polymorphism Q141K significantly reduces the ABCG2 expression, especially on the cell surface, because of a retention in the ER and increased degradation of the proteins (see below).

The Q141K variant has been found to significantly affect the toxicity of endogenous ABCG2 substrates, as well as the pharmacokinetics and toxicity of chemotherapeutic agents [28,55]. The first indication that this variant is associated with a major disease was recognized by genome-wide association (GWA) studies in gout patients. In fact, this finding led to the recognition of uric acid as an important endogenous substrate of this transporter [56]. The reduced membrane expression of Q141K-ABCG2 causes hyperuricemia and gout by a reduced excretion of uric acid, especially in the intestine by this transporter [57]. According to functional studies, Q141K-ABCG2 has lower transport capacity than the WT, thus, individuals who are homozygous for the alleles coding for this variant may have about 50% reduction in intestinal transport capacity of ABCG2 [52,57].

According to a guide document by the International Transporter Consortium [58], GWA, pharmacokinetic, and in vitro studies all support the clinical importance of this Q141K-ABCG2 variant. The blood area under the curve (AUC) level of sulfasalazine, an ABCG2 substrate, is greatly increased in patients carrying this variant [59], and altered plasma levels of—and clinical response to—other ABCG2 substrates, including atorvastatin, simvastatin, fluvastatin [60], sunitinib [61], or gefitinib [62], have also been reported. In addition, allopurinol (or its metabolites), applied for reducing hyperuricemia and treating gout, is also a substrate of ABCG2, thus, patients carrying a reduced function ABCG2 variant may have reduced response to allopurinol [63].

The allele resulting in the Q141K variant is more frequent (27–35%) in the Japanese and Chinese than in the Caucasian populations (6–14%), or in African American individuals (1–4%). This higher allele frequency makes the East Asian populations more vulnerable to the development of gout, or the toxic side effects of certain drugs (e.g., increased risk of rosuvastatin-induced myopathy [64,65]). This risk is even further increased by the relatively frequent presence of a mutation resulting in an early stop codon in the *ABCG2* gene (Q126X) in Asian populations [66].

Based on the importance of ABCG2 in human diseases and pharmacology, numerous reports have dealt with the biochemical background of the membrane expression and trafficking of the mutant and polymorphic variants, especially regarding the key elements of the biosynthesis, dimerization, glycosylation, and folding, as well as the surface appearance of the protein. ABCG2 is 655 amino acids long, and has molecular masses of 55 kDa and 75 kDa, with and without glycosylation, respectively. The protein has to homodimerize to create the active transporter [67,68], and an intermolecular disulfide bridge between cysteine 603 amino acids in the monomers stabilizes this dimer formation [67]. Interestingly, there is no major effect on the expression or processing of the transporter if cysteine 603 is mutated, and this intermolecular disulfide bridge cannot be formed. In contrast, the formation of intramolecular disulfide bridges within the monomers (involving C592 and C608) is required for the proper expression and folding of ABCG2 [67,68]. In the absence of the C592/C608 bond, most of the misfolded protein, after removal from the ER by retrotranslocation to the cytosol, is ubiquitinated and subsequently degraded in the proteasome.

ABCG2 has one effective N-glycosylation site (N596), which is normally fully glycosylated. However, there is relatively minor effect on ABCG2 expression, trafficking, or function by the removal of this glycosylation site [69]. Still, one careful study has shown that the non-glycosylated ABCG2 is more susceptible to ubiquitination and proteasomal degradation [70].

The cellular processing of the most common polymorphic variant, Q141K-ABCG2, has been studied in detail by several groups. Q141K decreases the ABCG2 protein expression and the localization of the protein to the plasma membrane [55,71,72,73,74,75,76], and such a reduction of expression also occurs in the red cell membrane [50]. Most probably, the transporter function of the protein is also slightly impaired, as the Q141K-ABCG2 protein shows a reduced ATPase activity in isolated membrane vesicles [55]. Still, as a main factor, the Glu to Lys amino acid change leads to an instability of the ABCG2 protein and an increased ER-mediated proteasomal degradation [72]. It has also been reported [73] that especially in overexpression systems or when the proteasomal degradation is inhibited, the protein is partially localized to intracellular aggresomes. Moreover, RNA regulatory mechanisms may also contribute to the reduced levels of the Q141K variant [74].

An improved folding and trafficking of the Q141K-ABCG2 can be achieved by small molecular correctors, including the histone deacetylase inhibitor 4-PBA [72]. Bafilomycin A1 treatment, reducing the lysosomal degradation, was also reported to increase the membrane levels of both the wild-type and the Q141K protein [75]. Colchicine is commonly used as a treatment for gout attacks, and this compound also has a positive effect on the membrane expression of this improperly folded variant, most probably through affecting microtubule formation, and especially the transport to aggresomes [76]. Recently, our group described a relatively frequent new missense variant of the ABCG2 (M71V), which has a similar but even more pronounced folding and trafficking problem than the Q141K protein. The addition of 4PBA or colchicine also improved the in vitro rescue and membrane expression of this variant [77].

**Figure 1:**
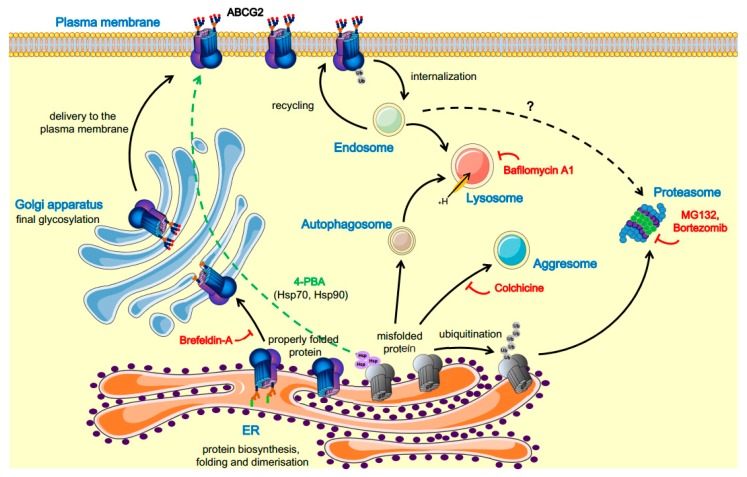
Schematic representation of the ABCG2 trafficking pathways and the respective modulators. The ABCG2 protein is synthesized on ER-bound ribosomes; dimerization and core glycosylation occur in the ER. The protein then travels to the Golgi complex, where its glycosylation is completed; thereafter, the mature ABCG2 travels to the plasma membrane. In contrast, the misfolded ABCG2 protein can be degraded by several pathways, including the lysosomal or the ubiquitin-mediated proteasomal degradation, as well as by accumulation in aggresomes. The misfolded forms caused by mutations can be rescued by inhibition of the degradation pathways or using pharmacological chaperones [1,4,70,73,75,78,79]. Figure 1 was prepared using image vectors from Servier Medical Art (www.servier.com), with licenses under the Creative Commons Attribution 3.0 Unported License (http://creativecommons.org/licenses/by/3.0/).

A schematic representation of ABCG2 trafficking pathways, including some drugs and modulators, is shown in Figure 1.

Based on the most recent analyses [71,80,81], we suggest that the numerous naturally occurring missense ABCG2 variants can be classified into three main groups. The first group of variants (e.g., the frequent V12M polymorphism or the naturally occurring D620N and K360del variants) result in the expression of fully functional ABCG2, with unchanged (or even somewhat increased) plasma membrane appearance, stability, and activity. In the second group, the ABCG2 protein function is only slightly affected by the mutation, but due to improper folding, they have a reduced level in the plasma membrane and higher degradation rate through the proteasomal or lysosomal routes. These variants, including the Q141K polymorphism and the rare M71V mutation, are good candidates for trafficking correction by small molecules. The third group of the ABCG2 variants, caused by splice site, nonsense, or frameshift mutations, comprises truncated proteins. These have a major structural and folding problem, and thus undergo immediate degradation at the central quality control. Examples for this third group include the Q126X (frequent in Asia), the Q236X and L264LhfsX14 (frequent in Europe), as well as the R383C mutants. These variants are responsible for the lack of the ABCG2 protein in the plasma membrane (in red cells leading to the Jr^–^ blood group [31]).

## 3. Potential Regulatory Regions within ABCG2 Affecting Processing and Trafficking

Atomistic protein structures can greatly help our understanding of the effects of amino acid changes in protein structure and function, and the high-resolution, atomic level structure of most parts of the ABCG2 protein is already available [82,83].

The functional unit of ABCG2 consists of two transmembrane and two nucleotide binding domains (Figure S1), similar to other ABC proteins, while the structural organization of domains in the ABCG subfamily members, termed Type II exporter structures, is substantially different from Type I exporters (which include ABCB1 or ABCC7). Since the ABCG2 TMD is short, the intracellular TM helices end directly at the intracellular leaflet of the bilayer, thus the coupling between nucleotide binding domain (NBD) and TM is in an intimate, close proximity of the membrane. Therefore, the crossing-over of the intracellular TM helices with the opposite NBD cannot occur, and the “domain swapping” characteristics of Type I exporters are not observed in the Type II exporter structures. The interface between NBD and TMD is also differently organized. The CFTR F508 residue is located at the boundary of a helix (H3) and a loop, interacting with one of the crossed-over intracellular loops (IL4). In contrast, the homologous ABCG2 F142 is in the middle of the elongated H3 helix and interacts with the connecting helix, TM1a [83], an amphipathic helix structurally analogous to the elbow helix in Type I exporters.

**Figure 2:**
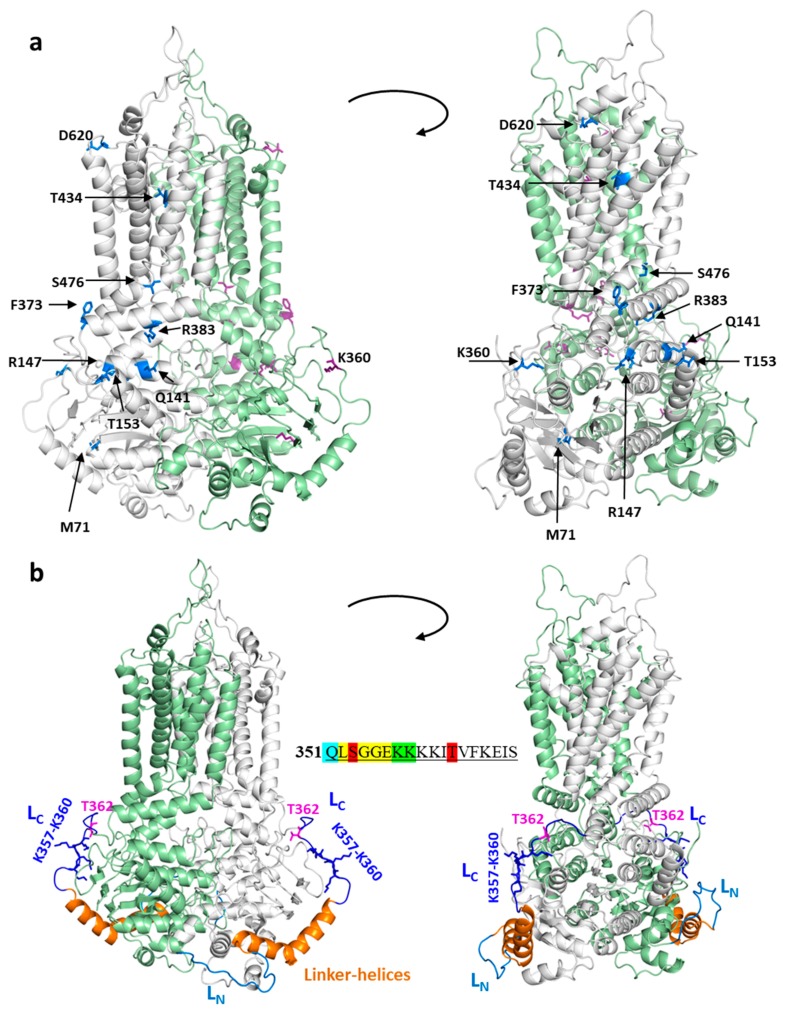
The localization of mutations and linker regions in the context of the ABCG2 structure. (**a**) Mutations are shown in blue and by stick representation in the bottom-closed conformation (PDBID:6HZM). The two ABCG2 protomers are colored in grey and green, respectively. (**b**) The N- and C-terminal loops (L_N_ and L_C_) are blue and dark blue, respectively. The resolved Linker-helices between these two loops are shown in orange. Stick representation is used for the T362 phosphorylation site and the four-lysine stretch. The sequence of this region is shown in the center. S353 and T362 were shown to be phosphorylated (red), K357 and K358 (green) were shown to be ubiquitinated, while the members of the LSGGE sequence, resembling the ABC signature (yellow), has been experimentally investigated. Q351 (blue) belongs to the helical structure.

When examining the effects of various mutations in the ABCG2 structural model (see Figure 2a), it has been shown that the naturally occurring mutations within important structural regions indeed cause major folding and processing errors. For example, T153M, Q141K, R147W, F373C, R383C, and S476P cluster together in or around the connecting helix, important for the communication between the NBDs and the TMDs. The effects of T153M and Q141K may be mild, since their side chain is exposed to the water phase, and the allosteric communication between domains is minimally altered. In contrast, R383, together with R382, has a crucial role, straddling the delocalized electrons of the F142 aromatic side chain (homologous to F508 in the CFTR), and thus stabilizing the interaction between NBD and TMD. This stabilization is abolished by the replacement of R383 by His or Cys (naturally occurring), or by Ala, Gly, and Lys (artificial) [79]. R147 forms a strong electrostatic interaction with E199, and this contact is ceased by a replacement of arginine by tryptophan. The effect of the M71V variants, causing improper folding and trafficking, could also be explained by the structural localization of this amino acid (see Zambo et al. [77] and Figure 2a).

In the atomic level structure of ABCG2, however, a relatively large region (from aa. 300 to 370) has not been resolved completely, while experimental data indicate a special role for intrinsically disordered loops located in this linker between the nucleotide binding and transmembrane domains. Interestingly, from L352 to E356, there is a consensus-like sequence of an ATP-binding signature region (L/VSGGE/Q—see Figure 2b insert), in addition to a verified signature sequence (VSGGQ) within the NBD domain of the human ABCG2. This bioinformatic resemblance stimulated the experimental and structural analysis of this region by Macalou et al. [84], revealing that residues L352 and S353 are important for the proper expression and function of ABCG2, while G354, G355, and E356 can be replaced by alanines, without any major effect on this protein.

S353 has been shown to be phosphorylated by mass spectrometry [85], while T362 in this region has been suggested to be the target of PIM-1 kinase-dependent phosphorylation [86]. According to this report and a following paper [87], this PIM-1 kinase phosphorylation has a major regulatory effect on the expression and membrane localization of ABCG2. In addition to these potential phosphorylation sites in this loop region, there are data available for the ubiquitination of ABCG2 in this loop from two large-scale studies. In human cells, Akimov et al. [88] revealed the ubiquitination of lysine 357, while in murine cells, a lysine corresponding to human K358 was shown to be ubiquitinated by Wagner et al. [89]. As mentioned above, the naturally occurring K360del mutation results in an unchanged or even increased plasma membrane expression for the ABCG2 protein.

In order to obtain potential structural information, we performed an in silico analysis of this linker region (a.a. 300–372). This loop contains a well-defined, central, V-shaped region (a.a. 326–353), which we name Linker-helices, with two shorter unresolved N- and C-terminal loops (L_N_ and L_C_, with boundaries 311–325 and 354–367, respectively). The L_N_ and L_C_ can either lack any secondary structure or possess stable structures with short flexible hinges, which render these regions mobile.

In order to gain insight into the structural background of mutations located in these loops, we modeled their structure using Modeller [90,91] and selected the models with the bests DOPE (Discrete Optimized Protein Energy) scores (Figure 2b). None of the loops exhibited parts with well-defined secondary structures. We also performed sequence-based JPRED (Protein Secondary Structure Prediction Server) secondary structure prediction [92], without using information from ABCG2 structures to confirm and strengthen the validity of these models. JPRED detected the last helix of NBD, the Linker-helices, and the connecting helix before the first TM helix, and indicated no secondary structure for the L_N_ and L_C_ regions (Figure S2).

The distance between the ends of the missing regions (L_N_ and L_C_) are similar (19.5 Å and 24.5 Å, respectively), as well as the number of spanning amino acids (15 and 14, respectively). However, L_C_ is likely more constrained compared to L_N_, since this C-terminally located loop has to avoid clashes with other parts of NBD, while connecting the two ends. In addition, the C-terminus contains hydrophobic amino acids (F364, Y369), which confine that end of L_C_ to the proximity of the membrane. A stretch of four lysines (K357–K360) may also contribute to this confinement via electrostatic interaction of their positive side chains with negatively charged lipid head groups. To strengthen this observation, we performed a molecular dynamics simulation using the full length ABCG2 structure with the modeled loops embedded in a lipid bilayer. The root mean square fluctuation (RMSF) of these loops reveals higher mobility for L_N_ compared to L_C_ (Figure S3). Importantly, the T362 and the Lys stretch (K357–K360) can be observed in the close proximity of the intracellular boundary of the membrane bilayer (Figure S3).

Taking the above results and considerations into account, two possible mechanisms of the unchanged or even increased expression caused by K360 deletion can be envisioned. First, the shortening of L_C_ together with the loss of a positive charge may weaken the interaction of the loop with the membrane bilayer, supporting an alternative conformation that may serve as a positive exit signal. Second, a retention signal may be disrupted by the deletion. Interestingly, this region, lacking a secondary structure, may include a degron sequence, although examining this ABCG2 sequence using the Eukaryotic Linear Motif (ELM) resource [93] revealed only one degron sequence associated with cell cycle (APCC activator-binding ABBA motif, Table S1). In addition, in this region, several sorting- and SUMOylation-related patterns were also identified. Although further in silico studies and experiments are required to determine the mechanism of altered expression upon mutations and the existence of a novel degron in this region, the matched ELM patterns (e.g., LIR motif that binds to Atg8 protein family members, SH2 domains binding motif, inverted version of SUMOylation motif, etc.) strongly suggest an important role of the linker region in sorting- and degradation-associated cellular processes.

Interestingly, the Pim1 phosphorylation site, T362, is also located in this region close to the lipid head groups. We speculate that the phosphorylation may introduce a large repulsion between the negatively charged phosphate and lipid head groups, and that this loop gains an alternative conformation similar to that of K360del, resulting in an increased functional expression of ABCG2. The experimental validation of these potential interactions is still needed.

## 4. Conclusions

Mutant variants of the human ABC membrane transporters often cause diseases or increase disease susceptibility due to impaired processing and trafficking. The ABCG2 protein is involved in endobiotic and xenobiotic transport, thus processing and trafficking mutants of this protein may increase the susceptibility to gout and also increase drug toxicity. We provide an overview and structural modeling for the disease-related polymorphic and mutant ABCG2 variants, focusing on the in silico analysis of a specific loop region with an as yet unresolved structure.

## 5. Methods

### 5.1. In Silico Methodologies

Structure: The PDBID:6HZM ABCG2 structure [83] was used for visualization, loop modeling, and molecular dynamics. Structures were visualized in PyMOL (The PyMOL Molecular Graphics System, Version 2.0 Schrödinger, LLC.). Secondary structure prediction: The human ABCG2 sequence (UniProt:Q9UNQ0) was submitted to JPRED (http://www.compbio.dundee.ac.uk/jpred/) [92]. Searching PDB (Protein Data Bank) before the prediction was skipped to dismiss existing structural information on the linker and its surrounding regions. Loop modeling: The loop modeling algorithm of Modeller [90] was applied for a.a. 311–325 and a.a. 354–367. All other atom positions in the ABCG2 structure were fixed. Sixteen models were generated with the molecular dynamics (MD)-level option with very slow refinement. The model exhibiting the best DOPE score was selected for further studies. Molecular dynamics (MD): A 0.5 µs long MD simulation was performed, with the ABCG2 structure (PDBID:6HZM) being supplemented with the modeled loops and embedded in a bilayer built from various lipids. GROMACS (GROningen MAchine for Chemical Simulations) with the CHARMM36m force field was used to run the simulation [94,95]. Details are provided in the Supplementary Materials. Analysis was performed for the last 100 ns of the trajectory by employing GROMACS tools and the MDAnalysis Python package [96]. Detection of short linear motifs: The ABCG2 sequence (UniProt:Q9UNQ0) was submitted to the ELM resource (http://elm.eu.org/search.html) [93]. A full set of results can be accessed at http://hegelab.org/resources.html.

### 5.2. Data Availability

The datasets generated and analyzed during the current study are available from http://hegelab.org/resources.html and from the corresponding authors on reasonable request.

## Figures and Tables

**Table 1:** A list of ABC transporters mentioned in the text and related conditions.

ABC Transporter	Related Disease or Condition
ABCC7 (CFTR)	cystic fibrosis (CF), male infertility
ABCC6 (MRP6)	pseudoxanthoma elasticum (PXE)
ABCB11 (BSEP)	type II progressive familial intrahepatic cholestasis (PFIC-II), drug toxicity
ABCB4 (MDR3)	type III progressive familial intrahepatic cholestasis (PFIC-III), drug toxicity
ABCG2 (BCRP/MXR)	gout, cancer multidrug resistance, drug toxicity

## Supplementary Materials

The following are available online at https://www.mdpi.com/2073-4409/8/10/1215/s1. Figure S1: Structural organization of ABCG2; Figure S2: Secondary structure prediction by JPRED; Figure S3: Molecular dynamics indicates higher flexibility of L_N_ when compared to L_C_; Table S1: Selected linear short motifs identified in the ABCG2 sequence (http://elm.eu.org).

## Abbreviations

4-PBA	4-phenylbutyrate
ABC	ATP-binding cassette
AUC	area under the curve
CF	cystic fibrosis
CFTR	cystic fibrosis transmembrane conductance regulator
DOPE	discrete optimized protein energy
ER	endoplasmic reticulum
GRASPs	Golgi reassembly stacking proteins
GWA	genome-wide association
GWAS	genome-wide association study
MD	molecular dynamics
MRP	multidrug resistance protein
NBD	nucleotide-binding domain
PXE	pseudoxanthoma elasticum
RMSF	root mean square fluctuation
SUMO	small ubiquitin-like modifier
TMD	transmembrane domain
