# Supplementary material for: Cellular Processing of the ABCG2 Transporter—Potential Effects on Gout and Drug Metabolism
Source: Cells. 2019 Oct 8;8(10):1215. doi: 10.3390/cells8101215 (PMC6830335; doi:10.3390/cells8101215)
Supplement: Supplementary file 1 [file cells-08-01215-s001.pdf]

SUPPLEMENTARY MATERIALS

Cellular processing of the ABCG2 transporter – potential effects in gout, ADME-Tox and drug metabolism

Orsolya Mózner [1], Zsuzsa Bartos [1,3], Boglárka Zámbó [1], László Homolya[1], Tamás Hegedűs[2,3]*, and Balázs Sarkadi [1,2,*]

Figure S1: Structural organization of ABCG2 [83,84]**.** This ABC transporter is a homodimer. Each protomer consists of a transmembrane domain (TMD, gray and green) and a nucleotide binding domain (NBD, white and pale green). The allosteric effects of ATP binding and hydrolysis are communicated between the two domains via the so-called connecting helix (CH). The NBD and TMD are connected by a linker (L, orange and red), which include a V-shaped secondary element formed by two helices (Linker-helices). There is a short α-helix in its N-terminus, which does not seem to belong to NBD and is structurally homologous to a helix located after the last α-helix (H9) of NBD (either to H9b or to the regulatory C-terminal extension of CFTR NBD1). The linker is not fully resolved, and two loops are not visible in the cryo-EM structures (L_N and L_C, dotted red and orange lines). EL3: extracellular loop 3.

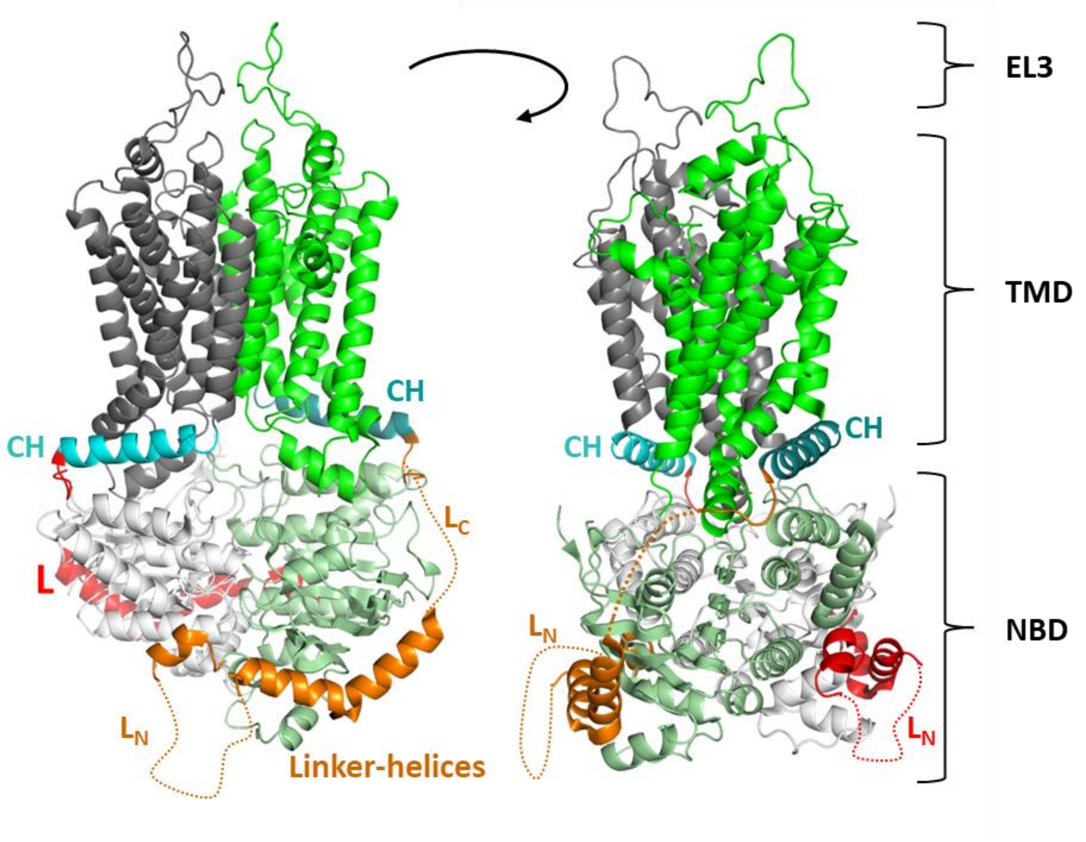

Figure S2: Secondary structure prediction by JPRED. The sequence of ABCG2 linker with neighboring helices was submitted to JPRED [94] for predicting its secondary structure without taking structures into account. The result matched the last α-helix (H9) from NBD1, the Linker-helices and the connecting helix, while the mobile, very small helix (S9b, a.a. 303-307) at the N-terminus of the linker was not detected. Importantly, JPRED did not indicate any secondary structure for L_N and L_C. This output strengthens the loop models provided by Modeller [92,93] (Figure 2).

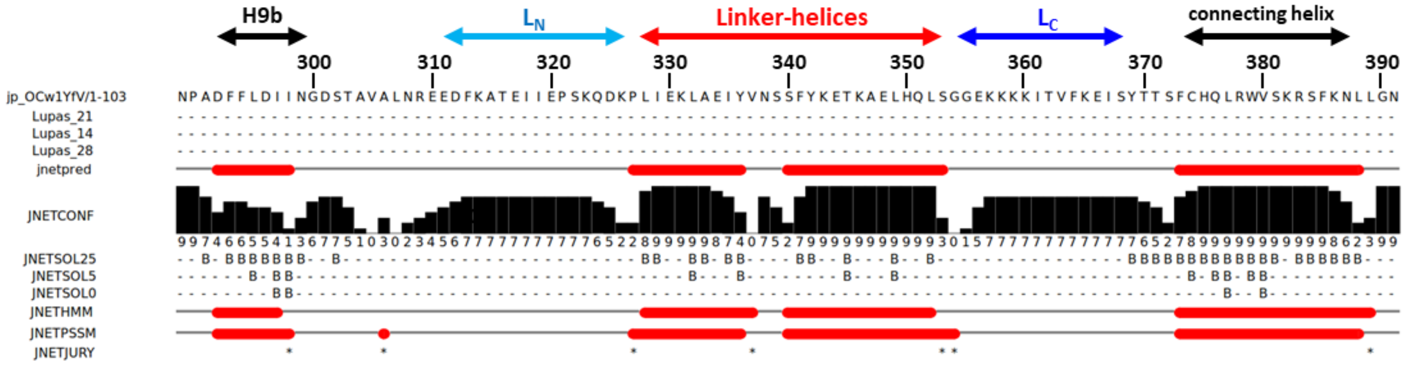

Figure S3: Molecular dynamics indicates higher flexibility of L_N when compared to L_C. An equilibrium molecular dynamics simulation was run for 0.5 μs using ABCG2 structure (PDBID: 6HZM, [84]) supplemented with the modeled loop regions. The protein was embedded into a complex lipid bilayer (outer leaflet: 25% POPC, 25% PLPC, 15% DPSM/SSM, 36% CHOL; inner leaflet: 18% POPC, 17% PLPC, 35% CHOL, 17% POPE, 8% POPS, 5% PIP2/DMPI25). The protein/lipid system was built using CHARMM-GUI ([97] http://dx.doi.org/10.1002/jcc.20945) and the simulation was performed using GROMACS ([96] see main text). Parameters for the energy minimization, the six equilibration steps and production run can be downloaded from http://hegelab.org/resources.html. Per residue root mean square fluctuation (RMSF) was calculated using GROMACS tools for the last 100 ns of the trajectory. RMSF values were inserted in the temperature factor column of the PDB file of the initial structure and depicted by PyMOL (The PyMOL Molecular Graphics System, Version 2.0 Schrödinger, LLC.). A thicker tube representation with warmer colors indicates higher flexibility (top panels). L_C exhibited lower flexibility compared to the N-terminal part and remained in contact with the main part of ABCG2. While the initial protein structure contained two different conformations of L_N in the two monomers, both symmetrical regions exhibited similar disengagement from NBD and became exposed during the MD simulation (left panel, front; right panel, left; bottom panel). During the simulation the membrane thickness increased when compared to the initial conformation, thus the coupling helices deepened into the bilayer. In addition, T362 and the stretch of the four lysines (K357-K360) are very close to the inner leaflet with the negatively charged lipid heads (bottom-panel).

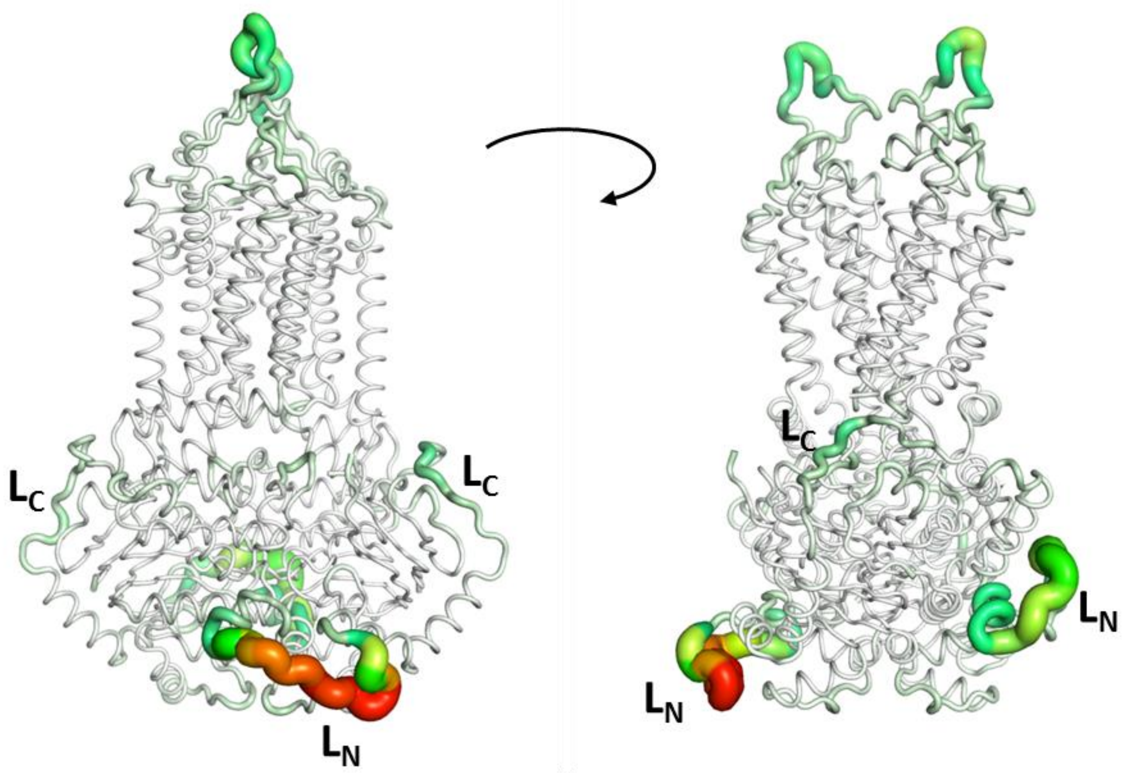

L_C
L_C
L_N
L_C
L_N
L_N

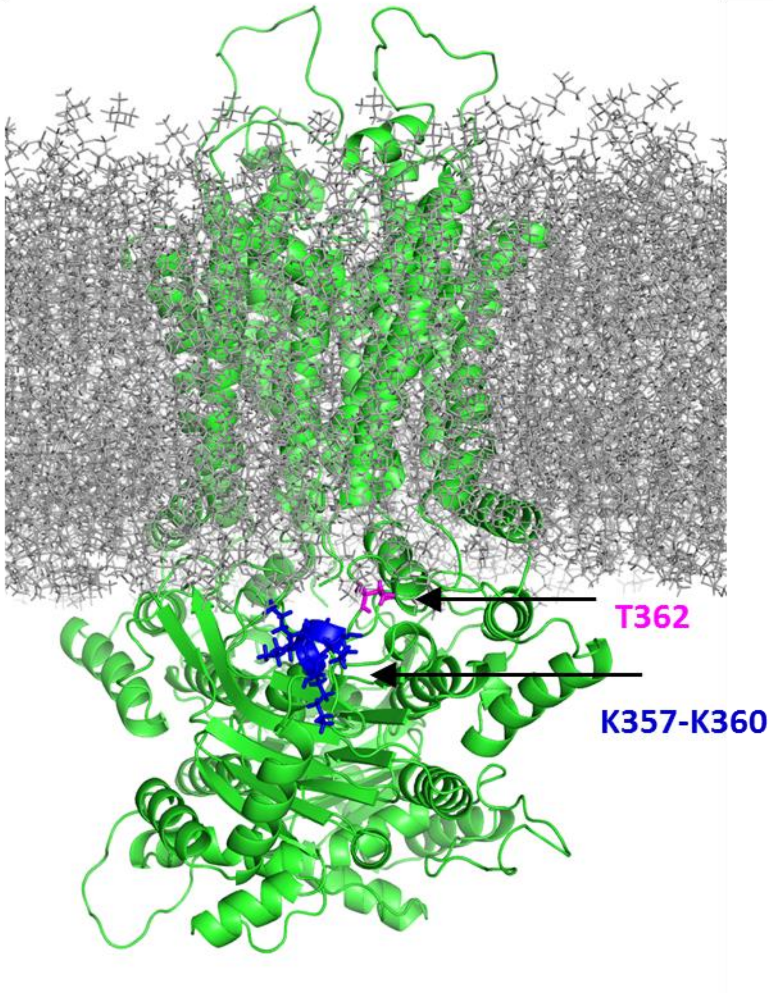

T362
K357-K360

Table S1: Selected linear short motifs identified in the ABCG2 sequence (http://elm.eu.org). A table with all matched entries can be found at http://hegelab.org/resources.html

Elm Name	Matched Sequence	Positions	Elm Description	Cell Compartment	Probability
DOC_MAPK_gen_1	KKKKITV	357-363	MAPK interacting molecules (e.g. MAPKKs, substrates, phosphatases) carry docking motif that help to regulate specific interaction in the MAPK cascade. The classic motif approximates (R/K)xxxx#x# where # is a hydrophobic residue.	nucleus, cytosol	0,004324
LIG_APCC_ABBA_1	ITVFKE	361-366	Amphipathic motif that is involved in APC/C inhibition by binding of CDH1/CDC20. In metazoan cyclin A, the motif also acts as a degron, enabling the cyclin's degradation in prometaphase.	spindle pole, nucleus, cytosol	0,0003843
LIG_APCC_ABBAyCdc20_2	KITVFKE	360-366	Amphipathic motif that binds to yeast Cdc20 and acts as an APC/C degron enabling cyclin Clb5 degradation during mitosis.	not annotated	0,0001669
LIG_FHA_1	KATEIIE	314-320	Phosphothreonine motif binding a subset of FHA domains that show a preference for a large aliphatic amino acid at the pT+3 position.	nucleus	0,008662
LIG_FHA_2	KETKAEL	343-349	Phosphothreonine motif binding a subset of FHA domains that have a preference for an acidic amino acid at the pT+3 position.	nucleus, Replication fork	0,008286
LIG_LIR_Gen_1	TVFKEI, SFKNL	362-367 384-388	Canonical LIR motif that binds to Atg8 protein family members to mediate processes involved in autophagy.	cytosol, cytoplasmic side of late endosome membrane	0,0052
LIG_PTB_Apo_2	IYVNSSFY	335-342	These phosphorylation-independent motifs bind to Dab-like PTB domains. Binding is not driven by contacts at the 0 or FY position, but instead is dependent upon the large number of hydrophobic and hydrogen bond contacts between motif and domain.	integrin,internal side of plasma membrane,cytosol,receptor complex,cytoplasmic membrane-bounded vesicle	0,0003108
LIG_SH2_GRB2	YVNS	336-339	GRB2-like Src Homology 2 (SH2) domains binding motif.	Early endosome, cytosol	0,0003019
LIG_SH2_SRC	YVNS	336-339	Src-family Src Homology 2 (SH2) domains binding motif.	cytosol	0,0008729
LIG_SH2_STAT5	YVNS, YTTS	336-339 369-372	STAT5 Src Homology 2 (SH2) domain binding motif.	cytosol	0,003296
MOD_PKA_1	KKITVFK	359-365	Main preference for PKA-type AGC kinase phosphorylation.	cAMP-dependent protein kinase complex, cytosol, nucleus	0,002315

MOD_Plk_1	VNSSFYK, KEISYTT	337-343 365-371	Ser/Thr residue phosphorylated by the Plk1 kinase	centralspindlin complex, nucleus, spindle, gamma-tubulin complex, midbody, cytosol, kinetochore, spindle midzone, nuclear condensin complex, cleavage furrow, nucleoplasm, microtubule organizing center	0,007674
MOD_Plk_4	VNSSFYK	337-343	Ser/Thr residue phosphorylated by Plk4	nucleus, cytosol, SCF ubiquitin ligase complex, cleavage furrow, centriole, gamma-tubulin ring complex, centriolar satellite, pericentriolar material	0,006019
MOD_SUMO_rev_2	EEDFKAT, SSFYKETKA	310-316 339-347	Inverted version of SUMOylation motif recognized for modification by SUMO-1	PML body, nucleus	0,0128
TRG_LysEnd_APsAcLL_1	QDKPLI	324-329	Sorting and internalisation signal found in the cytoplasmic juxta-membrane region of type I transmembrane proteins. Targets them from the Trans Golgi Network to the lysosomal-endosomal-melanosomal compartments. Interacts with adaptor protein (AP) complexes	cytosol,Endocytic vesicle	0,002758
TRG_Pf-PMV_PEXEL_1	KPLIE	326-330	Plasmodium Export Element, PEXEL, is a trafficking signal for protein cleavage by PMV protease and export from Plasmodium parasites to infected host cells.	endoplasmic reticulum, host cell cytoplasm, extracellular, host cell outer membrane, pathogen-containing vacuole membrane	0,002161
